# Mendelian randomization of immune cell phenotypes to discover potential drug targets for B-cell malignancy

**DOI:** 10.1038/s41408-025-01277-x

**Published:** 2025-04-09

**Authors:** Sina A. Beer, Molly Went, Charlie Mills, Codie Wood, Amit Sud, James M. Allan, Richard Houlston, Martin F. Kaiser

**Affiliations:** 1 Division of Genetics and Epidemiology, The Institute of Cancer Research, London, UK; 2 Department of Medical Oncology, Dana-Farber Cancer Institute, Boston, MA USA; 3 Broad Institute of MIT and Harvard, Cambridge, MA USA; 4 Harvard Medical School, Boston, MA USA; 5 Centre for Immuno-Oncology, Nuffield Department of Medicine, University of Oxford, Oxford, UK; 6 Translational and Clinical Research Institute, Newcastle University, Newcastle upon Tyne, UK

**Keywords:** Drug development, Cancer genetics

## Abstract

Although treatment options for B-cell malignancies have expanded, many patients continue to face limited response rates, highlighting an urgent need for new therapeutic targets. To prioritize candidate drug targets for B-cell malignancies, we employed Mendelian Randomization to estimate potentially causal relationships between 445 immune cell traits and six B-cell cancers: follicular lymphoma (FL), diffuse large B-cell lymphoma (DLBCL), Hodgkin lymphoma (HL), marginal zone lymphoma (MZL), chronic lymphocytic leukemia (CLL), and multiple myeloma (MM), totaling 22,922 cases and 394,204 controls. 163 traits showed a suggestive association with at least one B-cell malignancy (*P* < 0.05), with 34 traits being significant after correction for multiple testing (*P* < 2 × 10^−4^). By integrating findings with observational data and clinical trial evidence to support drug target candidacy, 24 cell surface markers were identified as druggable targets. In addition to established therapeutic targets such as CD3, CD20 and CD38, our analysis highlights BAFF-R and CD39 in HL, CD25 in MM, CD27 in CLL, CD80/86 in DLBCL, and CCR2 in FL and MZL as promising candidates for therapeutic inhibition. Our findings provide further support for the potential of human genetics to guide the identification of drug targets and address a productivity-limiting step.

## Introduction

Over the past decade, the treatment landscape for B-cell malignancies has been transformed with the introduction of new therapeutic agents [1, 2]. Despite these advances, many patients continue to experience treatment failure due to relapse or refractory disease [3, 4], leading to considerable challenges in delivering precision medicine and individualized treatment. Hence, there continues to be a need for new and improved treatments as well as prevention strategies for B-cell malignancies. As evidenced by recent FDA-approved drugs, targeting immune cell markers has been a highly successful strategy [5]. However, the attrition rate of drug development programs represents a productivity-limiting step [6]. The failure of preclinical model systems to adequately predict efficacy is leading drug developers to seek additional sources of evidence to inform decisions about which targets to pursue [7].

Genome-wide association studies (GWAS) have identified genetic variants associated with the levels of cell surface markers on specific subsets of immune cells [8], offering an opportunity to test the causal effect of potential drug targets using Mendelian randomization (MR). Briefly, MR capitalizes on genetic variation associated with modifiable exposures or risk factors to mitigate biases that affect traditional observational study designs. The approach uses genetic variants that are randomly assigned at conception as proxies for exposure to a risk factor, mimicking a randomized clinical trial [9]. Here, we performed a two-sample MR (2S-MR) to estimate the potential causal relationships between cell surface markers on subsets of immune cells and six B-cell malignancies, including follicular lymphoma (FL), diffuse large B-cell lymphoma (DLBCL), Hodgkin lymphoma (HL), marginal zone lymphoma (MZL), chronic lymphocytic leukemia (CLL), and multiple myeloma (MM). We integrated MR findings with observational and clinical trial evidence to support drug target candidacy.

## Methods

### Study design and ethical approval

**Fig. 1:**
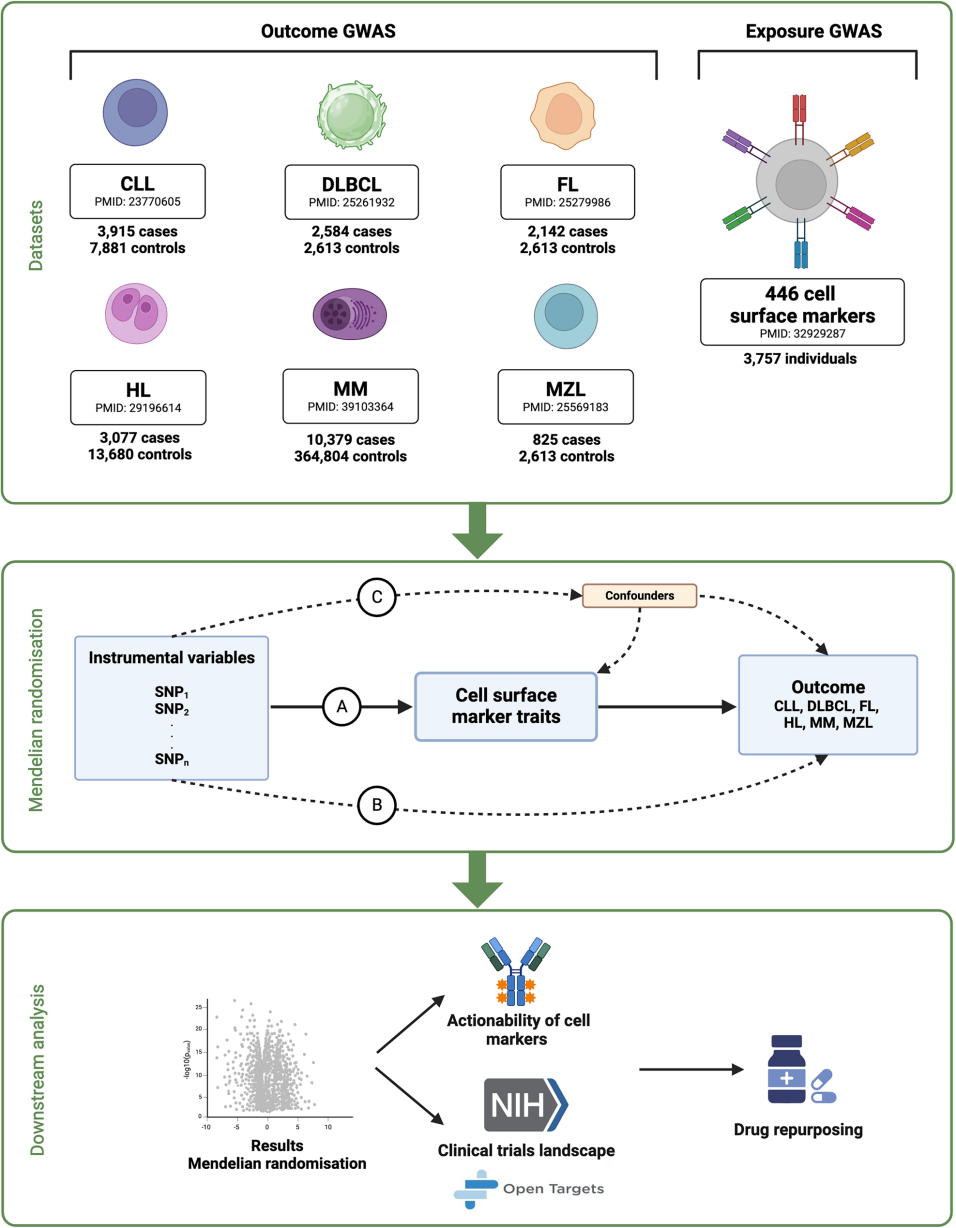
Study overview. Top panel: Overview of genome-wide association studies (GWAS) datasets used in the Mendelian randomization (MR) analysis. The outcome dataset comprised GWAS of six B-cell malignancies and the exposure dataset comprised one GWAS for immune cell traits. Case numbers and relevant publications are provided. Middle panel: Overview of MR, which relies on three key assumptions: (1) IVs must be independent of confounders, (2) associated with the exposure, and (3) influence the outcome solely through the exposure. Bottom panel: Downstream analysis following Two-Sample MR (2S-MR). To explore drug repurposing opportunities, associations were assessed for therapeutic actionability and their status in clinical trials. CLL Chronic Lymphocytic Leukemia, DLBCL Diffuse Large B-cell Lymphoma, FL Follicular Lymphoma, HL Hodgkin Lymphoma, MM Multiple Myeloma, MZL Marginal Zone Lymphoma. Figure created with BioRender.com.

We performed a 2S-MR of six B-cell malignancies, comprising 22,922 cases and 394,204 controls. Outcome data were derived from GWAS of six B‐cell malignancies: CLL, DLBCL, HL, FL, MM, and MZL. Exposure data were obtained from a GWAS that profiled immune cell traits using flow cytometry. A graphical summary of our study is shown in Fig. 1, which outlines the key elements and procedures deployed. In accordance with best practice, we adhered to the Strengthening the Reporting of Observational Studies in Epidemiology using MR (STROBE-MR) [10]. The analyses were based on published GWAS summary statistics (Fig. 1); hence no additional ethical approval was required for the study.

### Immune cell data and instrumental variable selection

We identified SNPs, serving as instrumental variables (IVs) from published studies by querying MR-Base using the data from Orrù et al. [11]. (Supplementary Data 10). For each SNP, the corresponding effect estimate on the trait expressed in *per* standard deviation (SD) unit change, and standard error (SE) was obtained. Only SNPs having a minor allele frequency (MAF) > 0.01 and a trait association *P*-value < 5 × 10^−8^ in a European population GWAS were considered. We excluded SNPs at high linkage disequilibrium (LD), imposing a threshold of r^2^ > 0.01, retaining SNPs with the largest effect. Duplicate exposure traits were manually reviewed and eliminated. Finally, we filtered the dataset of 614 traits to focus on immune cell traits characterized by the expression of one or more cell surface markers, excluding non-druggable traits associated with broader cell populations (*e.g*., lymphocyte absolute count), cell volumes, and side scatter (SSC) or forward scatter (FSC) parameters, resulting in a final set of 445 traits (Supplementary Table 1).

### Outcome data sources

To examine the association of IVs with risk of each B-cell malignancy we used GWAS summary effect estimates from the following sources: (1) Genotype data for CLL [12], MZL [13], DLBCL [14], and FL [15] studies were obtained from the database of Genotypes and Phenotypes (dbGaP) with accession code phs000801.v2.p1. These data were processed to derive summary statistics (see Supplementary methods). (2) For HL, MM and CLL, we used published data [16–19]. For HL and MM, we recomputed summary estimates excluding UK BioBank (UKBB) to avoid sample-overlap bias. Comprehensive information on each GWAS, including exclusion criteria, sex distribution, age at diagnosis as well as genotyping are available in primary publications (Fig. 1). In all cases we harmonized SNPs to ensure that the SNP effect estimates on exposure traits and malignancy risk were referenced to the same allele (Supplementary Data 11).

### Study power

The power of MR to demonstrate a relationship depends on the proportion of the variance explained (PVE) for each IV. To guard against weak instrument bias, we excluded any instruments with an F-statistic < 10. Study power for each target over a range of effect sizes was estimated as *per* Brion et al. stipulating a *P*-value of 0.05 [20].

### Phenotypes and genetic instruments

After harmonizing alleles and filtering out underpowered traits (F-statistic < 10), we analyzed on average 415 out of 445 immune cell traits per B-cell malignancy (range = 357–434), in conjunction with summary genetic data from published GWAS for CLL [12, 16, 17], DLBCL [14], HL [18], FL [15], MM [19], and MZL [13] (Fig. 1, Supplementary Table 1). The 445 traits were proxied by 1,745 genetic variants and the number of single nucleotide polymorphisms (SNPs) used as genetic instruments for each of the traits ranged from 1 to 37 (median: 2). The median PVE for each SNP used as an instrumental variable (IV) was 0.01%. Supplementary Fig. 1 provides an overview of hematopoiesis to assign immune cell phenotypes.

### Drug target analysis

To gain insight into the potential therapeutic value of targets identified we queried Open Targets [21], DrugBank [22], and ClinicalTrials [23]. We evaluated all classes of therapeutic agents, including small molecule inhibitors, monoclonal antibodies, and other biotherapeutics. These agents were evaluated based on their status in early or late-phase clinical development, preclinical models, and the strength of clinical or biological evidence supporting their activity in cancer. If no suitable agents were found, we expanded our search on available small molecules.

### Statistical analysis

For each SNP, effects were estimated for B-cell malignancy as an odds ratio (OR) per SD unit increase in the putative risk factor (OR_SD_). Depending on the number of SNPs serving as IV, we performed 2S-MR using the Wald ratio or inverse-weighted random effects (IVW-RE). We assessed the robustness of findings, using weighted median estimates (WME) and mode-based estimates (MBE) as well as applying a leave-one-out strategy to address potential biases from outlying and pleiotropic SNPs. Sensitivity analyses were performed using Cochran’s *Q*-test and Egger’s regression intercept and were considered significant if *P* < 0.05. To mitigate the potential impact of reverse association, we used MR Steiger to infer the direction of an effect, only retaining trait-outcome pairs with the correct direction, *i.e*., the trait is influencing the outcome and not vice versa (Supplementary Data 3-7). For this, we estimated the PVE using Cancer Research UK lifetime risk estimates for each of the six B-cell malignancies (Supplementary Table 3). Statistical analyses were performed using the TwoSampleMR package v0.5.6 [24] implemented in R (v3.4.0).

### Multiple comparison correction & assignment of statistical significance

After correcting for genetic correlations between traits by matrix decomposition using PhenoSPD [25], we identified 211 effectively independent phenotypes. With this in mind, we accounted for multiple comparisons by applying a Bonferroni correction, establishing statistical significance at a threshold of < 2.4 × 10⁻⁴ (*i.e*., 0.05/211). We considered associations at *P* < 2.4 × 10⁻⁴ as being statistically significant, while those with *P*-values between 2.4 × 10⁻⁴ and 0.05 as suggestive. We sought additional support for findings from *P*_WME_ or *P*_MBE_ < 0.05 (Supplementary Data 1) and confirmation of the predicted true causal direction via MR Steiger (Supplementary Data 4).

## Results

### Relationships between immune cell surface markers and B-cell malignancy

The relationship between each of the 445 genetically predicted immune cell traits and the risk of each B-cell malignancy is shown in Supplementary Data 1. Across all six B-cell malignancies, 163 unique immune cell phenotypes showed a suggestive association with disease risk (*P* < 0.05), including 32 phenotypes for CLL, 24 for DLBCL, 32 for FL, 63 for HL, 32 for MM, and 15 for MZL (Supplementary Table 2). Of the 163 observed associations, 34 were significant after correction for multiple testing (*P* < 2 × 10⁻⁴). The associations were not confined to B-cell-specific traits but also involved phenotypes related to T-cells and antigen-presenting cells (APCs). Of the 163 significant results, 34 immune cell phenotypes demonstrated cross-tumor effects with 11 of these having discordant associations between different B-cell malignancies (Supplementary Data 2, Supplementary Fig. 2). We confirmed the robustness of the associations by comparing causal estimates and associated *P*-values obtained from weighted median estimates (WME) and mode-based estimates (MBE) methods, with the Steiger test confirming the likely direction of causality (Supplementary Data 3–7).

**Fig. 2:**
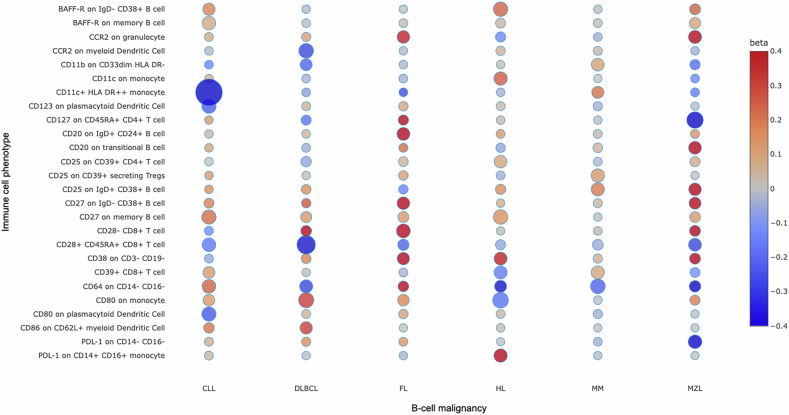
Bubble plot of selected immune cell traits showing a relationship with at least one B-cell malignancy. The columns correspond to the six B-cell malignancies. Bubble colors indicate the strength and direction of associations, with red corresponding to a positive association (i.e., higher expression linked to increased cancer risk) and blue corresponding to a negative association. Bubble size reflects the statistical significance of each association, measured by −log_10_ of the *P*-value, with larger bubbles denoting more significant results. *P*-values are unadjusted and two-sided. Empty cells denote missing data for the corresponding B-cell malignancy. Tregs T regulatory cells.

Table 1 provides an overview of all phenotype-defining cell surface markers with a relationship to at least one B-cell malignancy. Due to the large number of associations, we focused primarily on those where increasing surface marker levels were linked to a higher cancer risk, as these markers are more likely to be amenable to therapeutic intervention through inhibition. Selected discordant associations are presented to illustrate disease-dependent variability in cell surface marker expression (Fig. 2). As anticipated, we identified a potential causal relationship between traits related to an expanded B-cell subpopulation and a higher risk of B-cell malignancy. Genetically predicted increased levels of CD20 antigen-positive B-cells were associated with an increased risk of HL (suggestive, OR_SD_ = 1.45, 95% CI: 1.08–1.96, *P* = 1.49 × 10⁻²), FL (suggestive, OR_SD_ = 1.50, 95% CI: 1.03–2.19, *P* = 3.51 × 10⁻²), and MZL (suggestive, OR_SD_ = 1.52, 95% CI: 1.02–2.24, *P* = 3.74 × 10⁻²). Similarly, genetically predicted elevated levels of BAFF-R (B-cell activating factor receptor, alias TNFRSF13C), essential for B-cell survival and maturation, was associated with an increased risk of CLL (suggestive, OR_SD_ = 1.08, 95% CI: 1.01–1.15, *P* = 3.08 × 10⁻²), and HL (suggestive, OR_SD_ = 1.26, 95% CI: 1.06–1.49, *P* = 7.42 × 10⁻³). CD27 (alias TNFRSF7), a key marker of memory B-cells regulating immunoglobulin synthesis, was related to HL (significant, OR_SD_ = 1.19, 95% CI: 1.08–1.32, *P* = 7.07 × 10⁻⁴), CLL (suggestive, OR_SD_ = 1.24, 95% CI: 1.08–1.41, *P* = 1.58 × 10⁻³), DLBCL (suggestive, OR_SD_ = 1.21, 95% CI: 1.02–1.42, *P* = 2.41 × 10⁻²), and FL (suggestive, OR_SD_ = 1.50, 95% CI: 1.01–2.23, *P* = 4.32 × 10⁻²). CD38-mediated cancer risk depended on the underlying cell population. B-cells defined by higher CD38 expression were associated with a reduced risk of CLL (suggestive, OR_SD_ = 0.48, 95% CI: 0.25–0.91, *P* = 2.41 × 10⁻²), while higher CD38 expression on natural killer-enriched cells (CD3- CD19-) indicated an increased risk in HL (suggestive, OR_SD_ = 1.42, 95% CI: 1.03–1.95, *P* = 3.02 × 10⁻²).

**Table 1:** Genetically predicted increased immune cell phenotype levels and B-cell malignancy risk.

Cellular trait: potential drug target	Cell population	Effect of genetically predicted increased level of trait on B-cell malignancy risk.	
		Increased risk	Decreased risk
BAFF-R	B-cells / Plasma cells	CLL, HL	
CCR2	APCs		DLBCL, MZL
	Granulocytes	FL, MZL	
CD3	T-cells / Tregs	HL	DLBCL
CD11b	APCs / MDSCs	HL, MM	
CD11c	Granulocytes / APCs	HL	
	APCs		CLL, MM
CD14	MDSCs		FL
CD19	B-cells		HL, MM, MZL
CD20	B-cells	FL, HL, MZL	
CD25	B-cells	MM, MZL	FL, DLBCL
	T-cells / Tregs	HL, MM	DLBCL
CD27	Memory B-cells	CLL, DLBCL, FL, HL	
CD28	T-cells / Tregs		CLL, DLBCL, MM, MZL
CD34	HSC	FL	
CD38	B-cells		CLL
	CD3- CD19- cells	HL	
CD39	Tregs	HL, MM	DLBCL, MZL
	Granulocytes		MM
CD45RA	T-cells	MM	CLL, DLBCL, FL
CD64	APCs		DLBCL, FL, MM
	CD14- CD16- cells	CLL	
CD80	APCs	DLBCL	CLL, HL, MM
CD86	APCs	DLBCL	
CD123	APCs		CLL
CD127	T-cells		DLBCL, FL, MZL
CX3CR1	APCs		HL
HLA DR (A + B)	B-cells		FL
	T-cells		CLL, MM
	APCs	FL, MZL	DLBCL, CLL
PD-L1	APCs	HL	
	CD14- CD16- cells		MZL
TCRgd	T -cells	DLBCL, FL	HL

Displayed are 24 potentially druggable cell surface markers along with their expressing cell populations and the effect of genetically predicted increased levels of each marker on B-cell malignancy risk. The risk may either increase or decrease with higher levels of the respective marker. *APC* Antigen Presenting Cells, *Tregs* regulatory T cells, *MDSC* Myeloid-Derived Suppressor Cells, *NK* Natural Killer Cells, *CLL* Chronic Lymphocytic Leukemia, *DLBCL* Diffuse Large B-cell Lymphoma, *FL* Follicular Lymphoma, *HL* Hodgkin Lymphoma, *MM* Multiple Myeloma, *MZL* Marginal Zone Lymphoma.

Regarding T-cell and APC-related phenotypes across all B-cell malignancies, associations were frequently observed with costimulatory molecules or immune checkpoints (20.7%, Supplementary Table 1). Monocytes with genetically predicted higher levels of the immune checkpoint PD-L1 were linked to an increased risk of HL (suggestive, OR_SD_ = 1.61, 95% CI: 1.07–2.42, *P* = 2.20 × 10⁻²). Conversely, higher levels of PD-L1 on CD14- CD16- cells were associated to a decreased risk of MZL (suggestive, OR_SD_ = 0.39, 95% CI: 0.18–0.85, *P* = 1.81 × 10⁻²). Furthermore, the costimulatory interplay of CD28 on T-cells with CD80/86 on APCs is crucial for T-cell activation. In our analysis, increased levels of CD28 on CD28 + CD45RA + CD8 + T-cells were related to a reduced risk of DLBCL (significant, OR_SD_ = 0.69, 95% CI: 0.58–0.83, *P* = 7.26 × 10⁻⁵), CLL (suggestive, OR_SD_ = 0.85, 95% CI: 0.74–0.97, *P* = 1.63 × 10⁻²), and MZL (suggestive, OR_SD_ = 0.76, 95% CI: 0.58–0.98, *P* = 3.48 × 10⁻²), while higher levels of the CD8 + CD28- T-cell phenotype were linked to a risk increase in FL (suggestive, OR_SD_ = 1.79, 95% CI: 1.13–2.83, *P* = 1.31 × 10⁻²). Elevated expression of CD80 on monocytes and CD86 on dendritic cells (DCs) were both linked to an increased risk in DLBCL (suggestive, OR_SD_ = 1.34, 95% CI: 1.10–1.63, *P* = 3.56 × 10⁻³, and OR_SD_ = 1.34, 95% CI: 1.00–1.79, *P* = 4.85 × 10⁻², respectively). Conversely, higher CD80 expression on monocytes and DCs was linked to a reduced risk of HL (suggestive, OR_SD_ = 0.84, 95% CI: 0.75–0.94, P = 2.01 × 10⁻³) and CLL (suggestive, OR_SD_ = 0.79, 95% CI: 0.66–0.94, *P* = 1.00 × 10⁻²).

Genetically predicted levels of CD25 (alias interleukin-2 receptor, IL2Rα), which is partially influenced by the same genetic locus as CD28 and CD80, also contributed to the modulation of B-cell malignancy risk. The expression of CD25, in addition to CD4 and FOXP3, characterizes regulatory T cells (Tregs). Tregs play a critical role in defining tumor immune microenvironment (TME), especially in balancing immune tolerance [26]. Increased levels of CD3 on CD39+ Tregs were associated with higher HL risk (significant, OR_SD_ = 1.40, 95% CI: 1.17–1.67, *P* = 2.33 × 10⁻^4^). Further, higher levels of CD25 on CD39+ Tregs were linked to increased MM risk (suggestive, OR_SD_ = 1.13, 95% CI: 1.02–1.26, *P* = 2.16 × 10⁻²), but a reduced DLBCL risk (suggestive, OR_SD_ = 0.59, 95% CI: 0.38–0.91, *P* = 1.68 × 10⁻²). This observation is supported by activated B-cell populations with genetically predicted higher levels of CD25 being also associated with increased MM risk (suggestive, OR_SD_ = 1.21, 95% CI: 1.03–1.43, *P* = 2.44 × 10⁻²), and a reduced DLBCL risk (suggestive, OR_SD_ = 0.63, 95% CI: 0.44–0.92, *P* = 1.66 × 10⁻²). Beyond CD25, elevated levels of other IL receptors were consistently associated to reduced B-cell malignancy risk: CD127 (alias IL-7Rα) expression on T-cells was linked to a decreased risk in MZL (significant, OR_SD_ = 0.56, 95% CI: 0.40–0.79, *P* = 8.07 × 10⁻⁴), FL (suggestive, OR_SD_ = 0.60, 95% CI: 0.38–0.97, *P* = 3.53 × 10⁻²), and DLBCL (suggestive, OR_SD_ = 0.61, 95% CI: 0.39–0.95, *P* = 2.72 × 10⁻²), while CD123 (alias IL-3Rα) was linked to a lower risk in CLL (suggestive, OR_SD_ = 0.80, 95% CI: 0.68–0.95, *P* = 1.00 × 10⁻²).

Also, within the context of the TME, proinflammatory signaling is increasingly being recognized to be a critical factor in lymphomagenesis [27]. In keeping with this we observed associations between genetically predicted higher expression of CD11b and CD11c on monocytes with an increased risk of HL (suggestive, OR_SD_ = 1.12, 95% CI: 1.02–1.23, *P* = 2.20 × 10⁻², and OR_SD_ = 1.28, 95% CI: 1.04–1.59, *P* = 2.20 × 10⁻², respectively). Similarly, genetically predicted increased levels of CD11b on CD33dim HLA DR- cells, were associated with an increased risk of MM (suggestive, OR_SD_ = 1.11, 95% CI: 1.01–1.23, *P* = 3.62 × 10⁻²) and elevated levels of CD64 (alias immunoglobulin gamma Fc receptor I) on CD14- CD16- cells were linked to an increased risk of CLL (suggestive, OR_SD_ = 1.26, 95% CI: 1.05–1.53, *P* = 1.57 × 10⁻²). We also identified genetically determined elevated levels of the chemokine receptor CCR2 on myeloid DCs and on monocytes being coupled with reduced risk of DLBCL (suggestive, OR_SD_ = 0.76, 95% CI: 0.63–0.93, *P* = 6.12 × 10⁻³), and MZL (suggestive, OR_SD_ = 0.56, 95% CI: 0.34–0.92, *P* = 2.30 × 10⁻²), respectively. Conversely, higher CCR2 expression on granulocytes was linked to an increased risk of FL (suggestive, OR_SD_ = 1.42, 95% CI: 1.01–2.00, *P* = 4.18 × 10⁻²) and MZL (suggestive, OR_SD_ = 1.69, 95% CI: 1.06–2.68, *P* = 2.69 × 10⁻²).

### Drug actionability

**Fig. 3:**
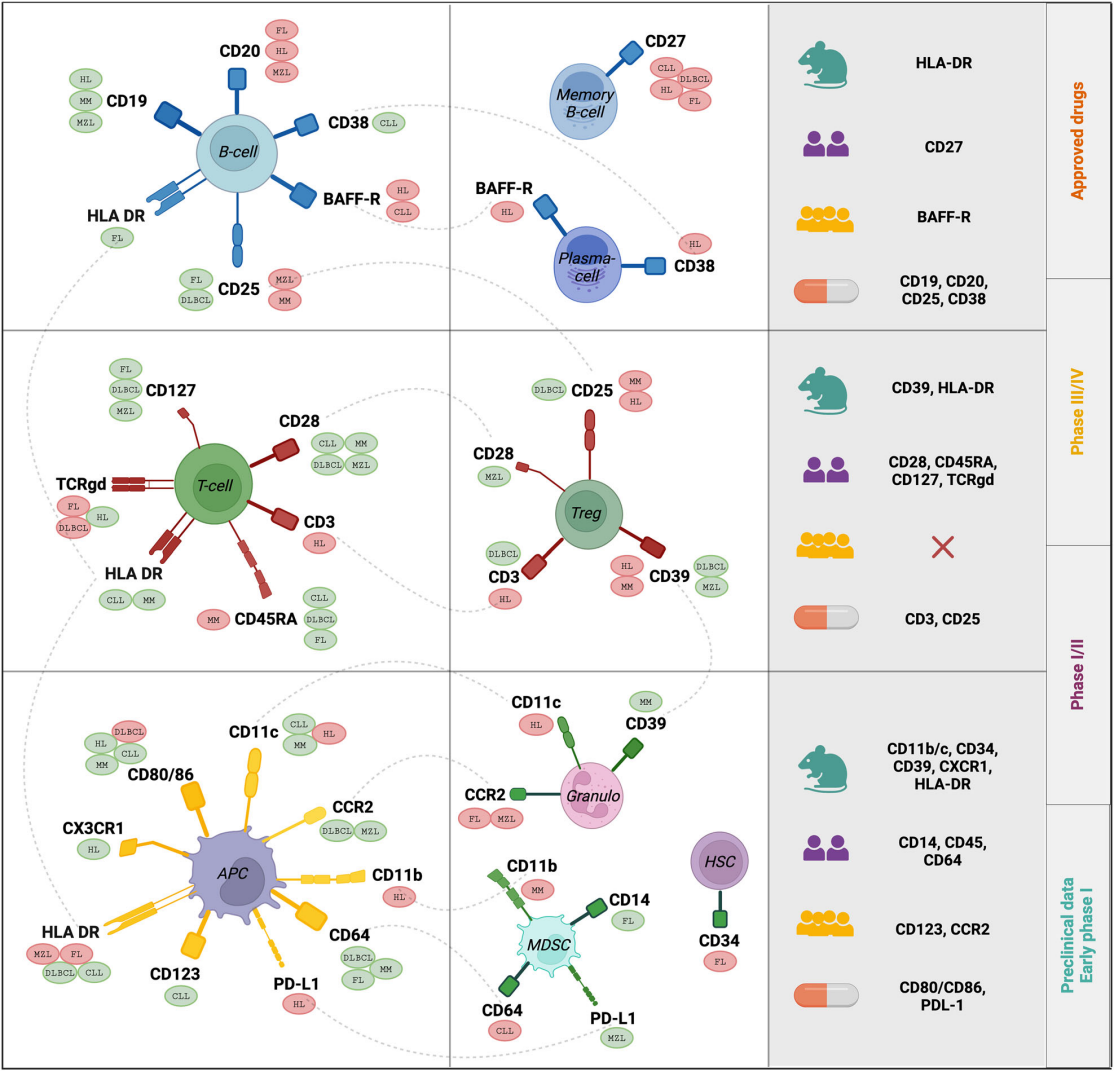
Cell surface markers showing a relationship with B-cell malignancy, categorized by cell type. The B-cell malignancy-associated risk for a specific cell surface marker is represented by a circle placed next to the marker. The color of the circle indicates the direction of risk. For genetically predicted higher levels of the cell surface marker red signifies an increased risk, while green denotes decreased risk. Additionally, the clinical trial status for each potential drug target is displayed on the right-hand side of the figure, with assigned colored symbols representing the corresponding trial phase (Data retrieved from OpenTargets.org and ClinicalTrials.gov). Figure created with BioRender.com.

**Fig. 4:**
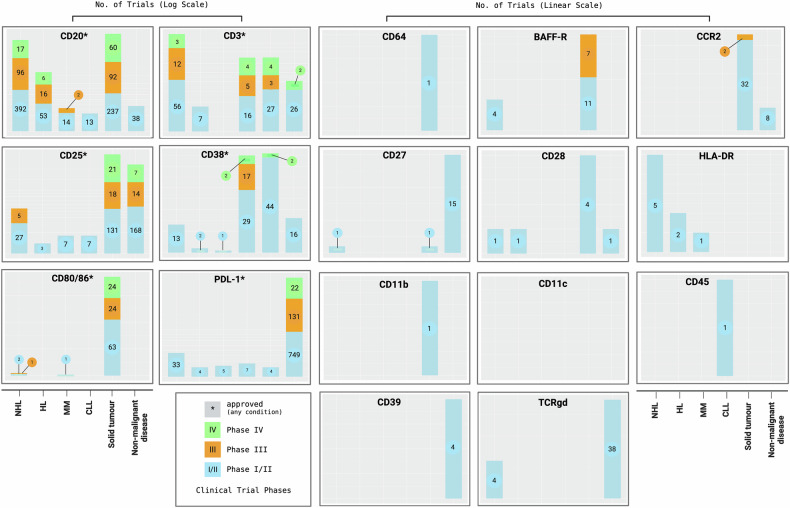
Clinical trial status for each cell surface marker identified as potential candidate for therapeutic inhibition. Each bar shows the clinical trial status of the cell surface marker as a drug target in B-cell malignancies as well as in solid tumors and non-neoplastic diseases. NHL (non-Hodgkin’s lymphoma FL, DLBCL, MZL), HL (Hodgkin lymphoma), MM (multiple myeloma), CLL (chronic lymphocytic leukemia). The left panel provides the status of six cell surface markers being targeted in advanced clinical trials (≥ Phase III) and which are approved for at least one condition. The right panel details the status of 12 markers which are primarily in early-phase trials. Created with BioRender.com.

We evaluated the clinical actionability of the identified relationships by referencing the Open Targets, DrugBank, CanSar, and ClinicalTrials databases. Of the 163 significant associations, we identified 24 (15%) cell surface markers as potentially druggable or suitable as drug targets (Table 1). Eighteen (11%) of these showed an association between increased genetically predicted expression and increased risk of at least one B-cell malignancy and hence potentially attractive candidates for therapeutic inhibition a priori (Fig. 3). Seven cell surface markers are the target of a currently approved therapy for B-cell malignancies, by means of monoclonal and bispecific antibodies (BiTEs), antibody-drug conjugates (ADCs), and CAR-T cells. These include CD3, CD19, CD20, and CD38, which we identified in our analysis. Our analysis also suggests BAFF-R and CD25 are likely to represent attractive drug targets. In keeping with such an assertion, it is noteworthy that both BAFF-R and CD25 are the subject of ongoing phase I/II clinical trials for relapsed/refractory (r/r) non-Hodgkin lymphoma (NHL) and r/r HL, respectively. Several additional cell surface markers that we identified, which may be plausible candidates for targeting in B-cell malignancy, have shown efficacy in treating solid tumors or autoimmune/inflammatory conditions (Fig. 4, Supplementary Data 8). These include, CCR2, CD27, CD80/86, as well as PD-L1, and represent as such a promising prospect for drug repurposing. Finally, our analysis highlights CD11b/c, CD64, and CD39. Although these markers are not currently under investigation (CD11b/c, CD64) or are only in early phase 1 trials for solid tumors (CD39), they remain potentially attractive targets, especially given the availability of pre-existing small molecule inhibitors.

## Discussion

Using 2S-MR, we have investigated the relationships between 445 immune cell traits and the risk of six B-cell malignancies. Our analysis identified 163 significant associations, including 24 unique cell surface markers, representing potential biomarkers or therapeutic targets. As well as providing insights into disease etiology, our findings serve to further illustrate the potential of human genetics to guide therapeutic target identification. By addressing productivity-limiting steps in drug development, human genetics overcomes bottlenecks to realize the vision of precision oncology [6, 28]. Many of the cell surface markers we identified as having a potentially causal relationship to B-cell malignancy risk are targets of already approved therapies in this field (*e.g*., CD3, CD20, CD38), or are under evaluation in solid tumors or autoimmune/inflammatory conditions (*e.g*., CD80/86, PD-L1). Our analysis serves to highlight potential avenues for therapeutic intervention including drug repurposing in B-cell malignancies.

Attractive targets for inhibition in B-cell malignancy are the costimulatory molecules CD27, BAFF-R, and CD80/86. Firstly, the assertion of targeting CD27 is supported by preclinical models demonstrating efficacy in targeting the CD27-CD70 axis, with a CD27-targeting mAb (CDX-1127) having recently entered clinical trials, albeit for solid tumors [29, 30]. Secondly, BAFF-R inhibition may be valuable in CLL and HL. Aside from seven ongoing Phase III trials in non-malignant conditions, BAFF-R inhibition is now the subject of a Phase I/II trial in NHL [NCT04903197]. Thirdly, targeting the costimulatory axis CD28 (T-cells) - CD80/86 (APCs) has just been subject of considerable interest as a therapeutic strategy with clinical trials being launched for a CD28×CD22 bispecific T-cell engager [NCT05685173] and a CAR-T cell product co-expressing kappa and CD28 in NHL/CLL [NCT04223765]. Our findings demonstrate that CD28 targeting may be an effective strategy, as increased levels of activated CD8 + CD28 + T-cells consistently correlate with a decreased risk of CLL, DLBCL, and MZL. In DLBCL, higher levels of CD80 and CD86 on APCs were strongly associated with an increased risk, which is particularly noteworthy given the well-established safety profile of CD80/86-targeting therapies from over 20 completed or ongoing clinical trials in inflammatory diseases. However, thus far, the only clinical trials targeting these molecules in B-cell malignancies were conducted over a decade ago, solely exploring inhibition in the context of HL [NCT00516217] and FL [NCT00117975] (Supplementary Data 8).

Tregs have a well-established role in autoimmune diseases by balancing immune tolerance [31], and our analysis revealed several shared immunological features to B-cell malignancies. It is intriguing that genetically predicted elevated levels of the Treg phenotype CD4 + CD25 + CD39+ were linked to increased risk of HL and MM in our analysis. This raises the prospect of invoking the ectoenzyme CD39 as a drug target for surface marker combinations. Preclinical models and early phase clinical trials have recently reported encouraging results in inhibiting this pathway in solid tumors, also in combining CD39 targeting with PD-1 immune checkpoint inhibitors [32]. Likewise, existing early-phase clinical trials in solid cancers and non-malignant conditions reinforce CCR2 inhibition, with targeting CCR2 on granulocytes showing promise for FL and MZL in our analysis. While we find at least some evidence for the above-mentioned drug targets, there is currently limited information on the integrin proteins CD11b and CD11c from clinical trials. However, given their association with increased HL risk and the availability of small molecules, they represent a promising target for future therapeutic strategies. A similar assertion may also apply to CD64 in the context of CLL.

While targeting APCs as a therapeutic strategy for treating some B-cell malignancies is in its infancy, our analysis supports such a rationale. Dual-targeting strategies engaging both tumor and T cells have shown promising effectiveness [33, 34]. Using ADCs, BiTEs, and CAR-T cells, there is the potential to devise combinatorial drug design strategies engaging APCs, targeting for example CD25 and CD39 in MM or CD25 and CD38 in MZL.

A major strength of our study is the agnostic approach for examining relationships between immune cell phenotypes and B-cell malignancies, limited only by the availability of suitable genetic instruments. 2S-MR minimizes concerns about confounding factors and reverse causation. Nevertheless, our study has the following limitations. Firstly, the sample sizes for MZL and FL were relatively small, with only 37% and 60% of phenotypes, respectively, achieving at least 80% statistical power (Supplementary Data 9), which inevitably restricted discovery for these malignancies. Secondly, the constraints of the immunophenotyping method by only identifying circulating cell surface marker proteins prevented us from evaluating all proteins within the same family or all proteins encoded by the same genomic region. Since the samples were derived from European populations, this may limit the extrapolation of our conclusions to other ethnicities. Finally, the failure of some past trials targeting the aforementioned molecules underscores that B-cell malignancies employ multiple mechanisms of immune escape, emphasizing the need to carefully consider both the combination and context of targeted therapies.

In conclusion, our MR study provides further insights into the etiological basis of six B-cell malignancies and while further experimental work is needed, several of the identified associations highlight promising candidates for biomarker development or therapeutic intervention. Since MR relations are associated with a higher likelihood of a particular target-indication pair being successful in drug discovery, we anticipate that in the future much larger-scale studies using additional proteins from a more extensive set of tissues will provide further insights informing drug development.

## Supplementary information

Supplemental Material

Supplementary Figure 2

Supplementary Data 1

Supplementary Data 2

Supplementary Data 3

Supplementary Data 4

Supplementary Data 5

Supplementary Data 6

Supplementary Data 7

Supplementary Data 8

Supplementary Data 9

Supplementary Data 10

Supplementary Data 11

## Data Availability

Genetic instruments can be obtained through MR-Base or from published work (Supplementary data 10). Summary GWAS cancer data are available from: Database of Genotypes and Phenotypes (dbGaP) with accession code phs000801.v2.p1. (CLL (US based study), MZL, DLBCL, and FL); GWAS Catalog ID: GCST000224 and GCST002299 (CLL, European based studies), GWAS Catalog ID: GCST007062 (HL); EGA under accession numbers EGA50000000280, EGAS50000000292, EGAZ50000000827, and EGAZ50000000828 [https://ega-archive.org/] (Multiple Myeloma).
