# Supplementary material for: Mendelian randomization of immune cell phenotypes to discover potential drug targets for B-cell malignancy
Source: Blood Cancer J. 2025 Apr 9;15(1):62. doi: 10.1038/s41408-025-01277-x (PMC11979003; doi:10.1038/s41408-025-01277-x)
Supplement: Supplementary file 1 — Supplemental Material [file 41408_2025_1277_MOESM1_ESM.pdf]

<u>Overview Supplemental Material</u>

SUPPLEMENTARY METHODS (page 2)

SUPPLEMENTARY TABLES (page 3)

Supplementary table 1: Details of filtering applied to instrumental variables used in the MR analysis.

Supplementary table 2: Total numbers of relationships per B-cell malignancy and per cell population.

Supplementary table 3: Lifetime risk of each B-cell malignancy (data from Cancer Research UK; https://www.cancerresearchuk.org/health-professional/cancer-statistics/risk/lifetime-risk; accessed October 2024).

SUPPLEMENTARY FIGURES (page 4)

Supplementary figure 1: Haematopoiesis overview.

Supplementary figure 2: Bubble plot of all immune cell traits showing a significant relationship with at least one B-cell malignancy.

SUPPLEMENTARY DATA (provided as separate Excel spreadsheets, legends page 5f)

Supplementary data 1: Causal estimates from the MR analysis for each trait and B-cell malignancy as well as a list of all 445 considered traits.

Supplementary data 2: Traits with significant relationships across the six B-cell malignancies categorised by cell population.

Supplementary data 3-7: Sensitivity tests for each exposure trait and B-cell malignancy. Few SNPs: WME/WM; Pleiotropy: MR-Egger; Steiger; Heterogeneity: IVW, Leave-One-Out.

Supplementary data 8: Identifiers for clinical trials registered for each potential drug target (data from OpenTargets.org and ClinicalTrials.gov).

Supplementary data 9: Power to demonstrate a causal association for each trait.

Supplementary data 10: SNPs serving as instrumental variables (IVs). Data from Orrù et al.

Supplementary data 11: Harmonised data for MR analysis for each trait and B-cell malignancy risk.

SUPPLEMENTARY METHODS

GWAS analyses of DLBCL, MZL, FL and CLL datasets. Standard quality-control measures were applied to each GWAS. Specifically, individuals with low SNP call rate (<95%) as well as individuals evaluated to be of non-European ancestry (using the HapMap version 2 CEU, JPT/CHB and YRI populations as a reference) were excluded. For apparent first-degree relative pairs, we excluded the control from a case-control pair; otherwise, we excluded the individual with the lower call rate. SNPs with a call rate < 95% were excluded as were those with a MAF <0.01 or displaying significant deviation from Hardy–Weinberg equilibrium ($P < 10^{-5}$). GWAS data were imputed to >10 million SNPs using IMPUTE2 v4 (for CLL) and IMPUTE2 v2.3 (for MM and HL) software [34, 35] in conjunction with a merged reference panel consisting of data from 1000 Genomes Project (phase 1 integrated release 3 March 2012) [36] and UK10K [37]. Genotypes were aligned to the positive strand in both imputation and genotyping. We imposed predefined thresholds for imputation quality to retain potential risk variants with MAF > 0.01 for validation. Poorly imputed SNPs with an information measure <0.80 were excluded. Tests of association between imputed SNPs and B-cell malignancy were performed under an additive model in SNPTESTv2.5. The adequacy of the case-control matching, and possibility of differential genotyping of cases and controls was evaluated using a Q–Q plot of test statistics. The inflation λ was based on the 90% least-significant SNPs and assessment of λ1000. Four principal components, generated using common SNPs, were included to limit the effects of cryptic population stratification in the CLL, DLBCL, FL and MZL data set. Eigenvectors for the GWAS data sets were inferred using smartpca (part of EIGENSOFT) by merging cases and controls with phase II HapMap samples.

---

34. Howie B, Marchini J, Stephens M. Genotype Imputation with Thousands of Genomes. G3 Genes|Genomes|Genetics. 2011;1(6):457-70.

35. Howie BN, Donnelly P, Marchini J. A flexible and accurate genotype imputation method for the next generation of genome-wide association studies. PLoS Genet. 2009;5(6):e1000529.

36. Abecasis GR, Altshuler D, Auton A, Brooks LD, Durbin RM, Gibbs RA, et al. A map of human genome variation from population-scale sequencing. Nature. 2010;467(7319):1061-73.

37. Huang J, Howie B, McCarthy S, Memari Y, Walter K, Min JL, et al. Improved imputation of low frequency and rare variants using the UK10K haplotype reference panel. Nat Commun. 2015;6:8111.

SUPPLEMENTARY TABLES

	CLL	DLBCL	FL	HL	MM	MZL
Starting no. of traits (SNPs)	614 (2.818)					
Manually filtering for cell surface marker traits (excluding cell counts / volumina, SSC/FSC parameters)	446 (1.745)					
Traits remaining after harmonisation	422	436	435	422	431	362
Traits with no missing info and Fstat >10	420	434	431	420	428	357

Supplementary Table 1 | Details of filtering applied to instrumental variables used in the Mendelian randomisation analysis. Final row (after all filtering steps) indicates number of traits analysed for each B-cell malignancy.

B-cell malignancy	CLL	DLBCL	FL	HL	MM	MZL
**Number of relationships**	**32**	**24**	**32**	**63**	**32**	**15**
B-cell related traits	18	6	11	18	9	4
	56%	25%	34%	29%	28%	27%
T-cell related traits	2	8	12	24	15	4
	6%	33%	38%	38%	47%	27%
APC related traits	9	7	6	18	4	4
	28%	29%	19%	29%	13%	27%
Other cell population related traits	3	3	3	3	4	3
	9%	13%	9%	5%	13%	20%

Supplementary Table 2 | Results of Mendelian randomisation analysis split up by B-cell malignancy and cell population. The total number of causal relationships per cancer is further categorised by cell population, including B-cell traits, T-cell traits, APC traits, and other cell populations (e.g., granulocytes, basophils). Alongside the absolute number of associations, the percentage of traits within each cell population per cancer is provided.

B-cell Malignancy	CLL	DLBCL	FL	HL	MM	MZL
Lifetime risk	0.01	0.005	0.005	0.005	0.01	0.005

Supplementary Table 3 | Lifetime risk estimates for each B-cell malignancy used to estimate the proportion of variance explained.

SUPPLEMENTARY FIGURES

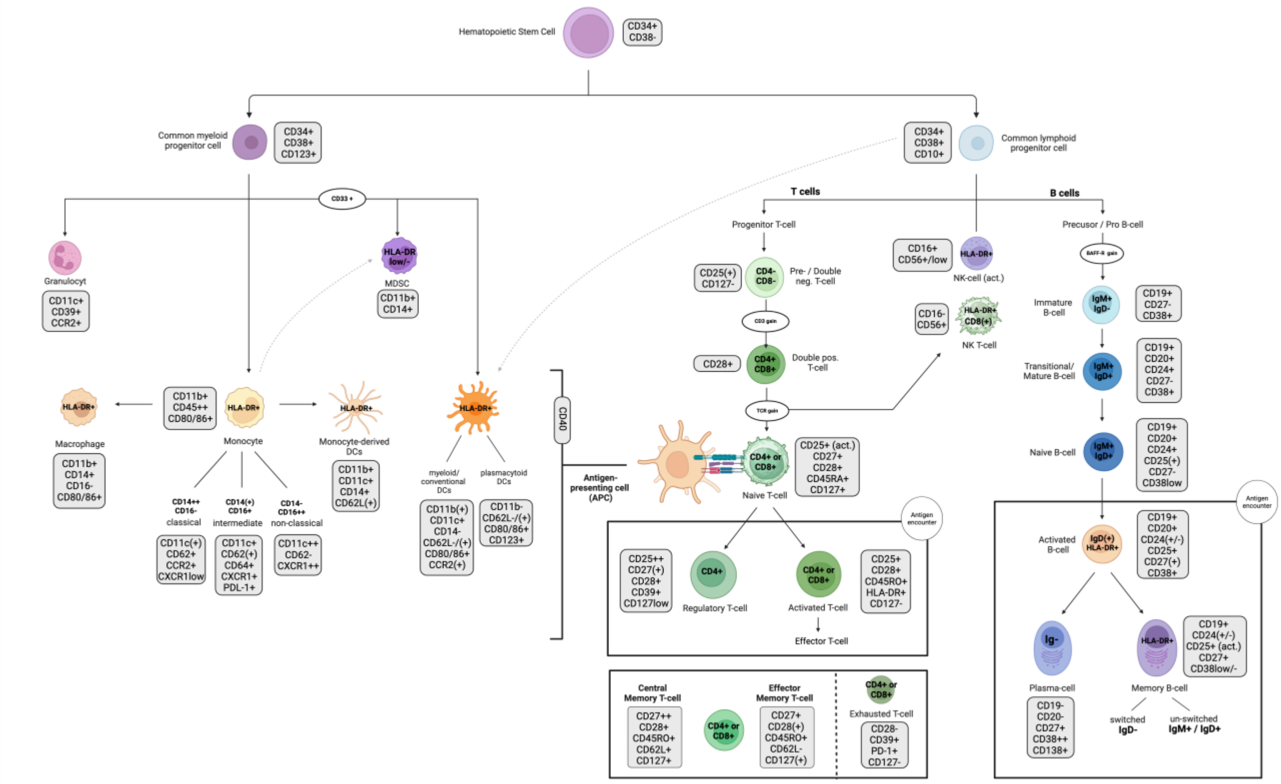

Supplementary Figure 1 | Haematopoiesis overview. Schematic overview of hematopoietic cell lineage differentiation from hematopoietic stem cells (HSCs) into myeloid and lymphoid lineages. APC: Antigen-presenting cell; DC: Dendritic cell; DN: Double negative; DP: Double positive; HLA-DR: Human leukocyte antigen – DR isotype; MDSC: Myeloid-derived suppressor cell; NK: Natural killer; Treg: Regulatory T cell.

Provided as supplementary data file due to the size of the figure.

Supplementary Figure 2 | Bubble plot of all immune cell traits showing a significant relationship with at least one B-cell malignancy. The columns correspond to the six B-cell malignancies. Bubble colours indicate the strength and direction of associations, with red corresponding to a positive association (*i.e.,* higher expression linked to increased cancer risk) and blue corresponding to a negative association. Bubble size reflects the statistical significance of each association, measured by $-\log_{10}$ of the *P*-value, with larger bubbles denoting more significant results. *P*-values are unadjusted and two-sided. Empty cells denote missing data for the corresponding B-cell malignancy. Tregs = T regulatory T cells; MDSC = Myeloid-Derived Suppressor Cells.

SUPPLEMENTARY DATA LEGENDS

Supplementary Data 1 | Causal estimates from the Mendelian randomisation analysis for each trait and B cell malignancy risk

Results reported as odds ratios (OR_{SD}) and 95% confidence intervals (CIs) per genetically predicted standard deviation (SD) unit increase in the risk factor. Two-sided *P*-value reported.
Wald ratio reported if only 1 SNP available for use in MR analysis. Inverse-variance weighted random effects model only computed if ≥2 SNPs available for use in MR analysis.
Additional support is defined by *P* WME or *P* MBE < 0.05 and predicted true causal direction using MR Steiger.
SNP, single nucleotide polymorphism; OR, odds ratio; LCI, lower 95% confidence interval; UCI, upper 95% confidence interval; N, trait sample size

Supplementary Data 2 | Immune cell phenotypes with significant relationships across six B-cell malignancies.

B-cell, T-cell, APC, and other cell-type related traits are listed on separate sheets.
The odds ratio per standard deviation (ORSD) is provided for each of the six B-cell malignancies per trait. In cases of a significant association, the field is colour-highlighted: green for an ORSD < 1 and red for an ORSD > 1. White fields indicate no significant results in the 2SMR analysis. Fields marked as NA indicate data not available.

Supplementary Data 3 | Weighted median estimate and mode-based estimates for each exposure trait and B-cell malignancy.

Results reported as effect size (beta) and standard error (SE) per genetically predicted standard deviation (SD) unit increase in the risk factor. Two-sided *P*-values shown.
Weighted median estimator and mode-based estimate only computed if ≥3 SNPs available for use in MR analysis.
SNP, single nucleotide polymorphism; SE, standard error.

Supplementary Data 4 | MR Steiger analysis for each exposure trait and B-cell malignancy.

Shown are two-sided tests for P-value. R2, proportion of variance explained; SNP, single nucleotide polymorphism; MR, Mendelian randomisation

Supplementary Data 5 | MR-Egger regression analysis for each exposure trait and B-cell Malignancy.

MR-Egger estimates only computed if ≥5 SNPs available for use in MR analysis. Shown are tests for two-sided P-values.
SE, standard error; N, trait sample size; MR, Mendelian randomisation.

Supplementary Data 6 | Leave one out inverse variance weighted random-effects MR analysis for each exposure trait and B cell malignancy.

Results reported as effect size beta and standard error (SE) per genetically predicted standard deviation (SD) unit increase in the risk factor. Two-sided P-value reported. Leave-one-out analysis not conducted if ≤2 SNPs available for use in MR analysis. Traits with NA indicate those with too few SNPs to perform leave-one-out analysis.
N, trait sample size; MR, Mendelian randomisation.

Supplementary Data 7 | Sensitivity tests for each exposure trait and B-cell malignancy.

Results reported as effect size (beta) and standard error (SE) per genetically predicted standard deviation (SD) unit increase in the risk factor. Two-sided P-values shown.
Sensitivity tests: few SNPs, pleiotropy, heterogeneity, and leave-one-out IVW analyses.
SNP, single nucleotide polymorphism; SE, standard error; N, trait sample size

Supplementary Data 8 | Clinical trial identifiers for trials registered for each potential drug target associated with a risk increase in 2SMR.
Clinical Trials were found on Opentargets.org (Sheet 1) or Clinical Trials.gov (Sheet 2).
The list includes the NCT number (trial identifier), clinical indication (the disease being studied), the clinical phase (I-IV), the trial status (ongoing, completed, recruiting, terminated), and if applicable, the reason for withdrawal or termination of the trial.

Supplementary Data 9 |Power for each trait considered in MR analysis.
Power for each trait calculated across a range of odds ratios.
SNP, single nucleotide polymorphism; OR_{SD}, odds ratio per standard deviation unit increase in the putative risk factor; N, trait sample size; R^2, estimate of proportion of variance explained

Supplementary Data 10 | SNPs serving as instrumental variables (IVs) from published studies by querying MR-Base using the data from Orru et al. *
SNP, single nucleotide polymorphism; SE, standard error; N, trait sample size; EAF, effect allele frequency; PMID, PubMed identifier. * [https://www.ncbi.nlm.nih.gov/pmc/articles/PMC8517961/]

Supplementary Data 11 | Harmonized Data for the Mendelian randomisation analysis for each trait and B cell malignancy risk
SNP, single nucleotide polymorphism; EAF, effect allele frequency; SE, standard error; Chr., chromosome; N, sample size; MAF, minor allele frequency; PVE, proportion of variance explained.
